# The molecules and mechanisms of heart development, disease and regeneration

**DOI:** 10.1186/1471-2164-15-S2-O21

**Published:** 2014-04-02

**Authors:** Eric N Olson

**Affiliations:** 1 University of Texas Southwestern Medical Center, Dallas, TX, USA; 2 Center of Excellence in Genomic Medicine Research (CEGMR), King Abdulaziz University, P.O. Box: 80216 Jeddah 21589, Kingdom of Saudi Arabia

## 

We seek to define the gene regulatory networks that govern heart development and disease. Recently, we discovered that the hearts of neonatal mice can fully regenerate after partial surgical resection or myocardial infarction, but this capacity is lost early in life. We are currently exploring the molecular underpinnings of the neonatal regenerative response of the heart, with the long-term goal of discovering combinations of genes and drugs that promote cardiac repair and regeneration. Promotion of cardiomyocyte proliferation through activation of the Yap pathway and modulation of epicardial signaling systems have shown efficacy in enhancing these processes. We are also optimizing strategies for reprogramming of cardiac fibroblasts toward a cardiac cell fate as a means of replacing cardiomyocytes in injured hearts. We have shown that four transcription factors can cooperatively reprogram fibroblasts into cardiac-like myocytes in vitro, albeit relatively inefficiently. Forced expression of these factors in dividing non-cardiomyocytes in mice also allows reprogramming into functional cardiac-like myocytes, improves cardiac function and reduces adverse ventricular remodeling following myocardial infarction. Screens for small molecules and microRNAs that enhance cardiac reprogramming have revealed new insights into the mechanistic basis of this process and have allowed further optimization in human cells. Opportunities and obstacles in the path toward mammalian cardiac regeneration will be discussed.

